# Taking stock: Can captive species contribute to biodiversity conservation?

**DOI:** 10.1371/journal.pone.0354602

**Published:** 2026-08-19

**Authors:** Justin J. Lennon, Angelo P. Pernetta, Rachel L. White, Neil Crooks

**Affiliations:** 1 School of Applied Sciences, University of Brighton, Brighton, United Kingdom; 2 National Marine Aquarium, Plymouth, United Kingdom; 3 Heron International, London, United Kingdom; 4 School of Animal, Rural and Environmental Sciences, Nottingham Trent University, Nottingham, United Kingdom; University of Minnesota, UNITED STATES OF AMERICA

## Abstract

Strategic collection planning is a central component of contemporary zoological institutions, yet its underlying motivations remain debated. Zoos are recognised as contributing to biodiversity conservation, although it is unclear whether individual collections reflect this role. As visitor-derived income is the primary revenue source for most zoos, collection planning may favour large, traditionally charismatic species that attract public interest, potentially creating tension between conservation objectives and financial sustainability. Previous studies have identified a bias towards non-threatened species, but these have largely relied on global threat classifications, such as the IUCN Red List, which may overlook species of regional or local conservation importance. Here, we assess the size and diversity of zoo populations across all vertebrate taxa held in UK institutions, comparing global threat status with the representation of native and locally prioritised species. Shannon Wiener diversity indices indicated that fish were the most diverse vertebrate class (H = 5.41), while amphibians were the least diverse (H = 3.72). Population sizes of globally non-threatened species were more than double those of globally threatened species. UK native and priority species accounted for 9% and 2%, respectively, of the total BIAZA collection, with both groups represented across all vertebrate taxa. Globally threatened species native to the UK were recorded only among fish and birds. Additionally, six non-native species classified as Extinct in the Wild were identified, comprising five fish species and one bird species. Although collection planning within BIAZA institutions remains biased towards globally non-threatened species and species non-native to the UK, these findings demonstrate the capacity of UK zoos to contribute to native and priority species conservation and underline the importance of maintaining comprehensive captive populations for regional management and globally shared taxonomic data.

## Introduction

Zoos and aquariums (hereafter zoos) have undergone a significant ideological and moral evolution throughout their history. Where the *raison d’être* of a zoo was primarily to entertain its visitors, their role has diversified to also encompass: conservation of the most threatened species [1]; protection and restoration of wild habitats [2–4]; education of the public on topics such as the biodiversity crisis; [5] and research and research training, including animal behaviour, husbandry and veterinary sciences [6,7]. These roles are often aligned with other important messages such as planetary health, human wellbeing and sustainable living [8]. This change in ethos is influenced by a desire for social relevance [9] and pressure from local governments to comply to standards for the purpose of zoo licensing [10]. A small but growing number now attempt to implement a *One Plan Approach*, where zoos work collaboratively with other organisations and wildlife managers to conserve biodiversity [11]. It is estimated that the global collective audience of zoos is around 700 million visitors annually [12]. Given the large volume of visitors that pass through these institutions every year, zoos provide a unique opportunity to educate people and aid biodiversity conservation [13]. Zoos however are continually criticised for providing inadequate living conditions, particularly for species that are non-native or require significant space to mimic their natural habitat [14]. The success and effectiveness of zoo conservation activities, such as *ex situ* breeding and reintroduction programmes are often questioned along with a lack of commitment towards *in situ* conservation [15]. However, the conservation roles of a contemporary zoo are diverse, and therefore assessing their contributions will require examination of their investment into education and research [16].

Zoos compete with other tourist attractions for visitors and therefore must provide entertainment experiences to appeal to their audience [17]. Puan and Zakaria [18] suggested that entertaining visitors is necessary in ensuring an effective learning experience especially when aimed at children and those who are visiting as part of a leisure experience. Zoos must strike a balance between articulating and evidencing their conservation efforts along with ensuring their facilities provide entertainment in order to maintain social relevance and a sustainable business model [9].

Contemporary zoos use their collections as ambassadors to help fulfil their educational obligations [19]. Communicating effectively the range of threats faced by wildlife requires a diverse collection of species from all major animal taxa. According to Eisenhauer and Hines [20] approximately 95.5% of all extant species are invertebrates. If zoos were to reflect biodiversity in proportions consistent with its occurrence in the wild, vertebrate taxa would comprise fewer than 5% of collections. However, it is unlikely that an assemblage of molluscs and insects would capture and retain the interest of the paying visitors and thus unrealistic for institutions to aspire to mirror *in situ* biodiversity accurately [21,22]. If the main driver for zoo collection planning was conservation, there would be an expectancy for them to house principally threatened species requiring further conservation assistance through research or breeding and release [14]. However, in instances where *ex situ* populations cannot be sustainably maintained and do not clearly contribute to recovery, housing non-threatened species maybe more ethical than housing threatened species since removal from the wild is less likely to worsen extinction risk [23–25].

The recent State of the World’s Amphibians report [26] found two in every five amphibian species to be threatened with extinction, making them the most threatened vertebrate class [27]; however, like invertebrates, collections of these individuals are unlikely to appeal to visitors [22]. Instead, zoos should attempt to represent a selection of all major vertebrate taxa (Mammals, Birds, Fish, Reptiles and Amphibians) allowing visitors to foster an understanding of the diversity of life and the threats they face in the wild [21]. This attempt to house a diverse collection of species often falls short, with zoos opting to showcase more charismatic, visitor ‘favourites’ that may contradict the other key principles of the organisation, such as breeding threatened species or providing conservation pedagogy [21,28,29]. This requirement to entertain visitors has resulted in a taxa bias towards mammals and birds over reptiles, amphibians and fish [30], as well as housing non-threatened species in higher numbers than those that are threatened [31]. Furthermore, captive species are less likely to share other characteristics associated with high conservation priority such as having restricted geographical ranges or being found in high-risk ecosystems [32].

Contemporary zoos utilise global threat-ranking criteria to inform conservation decisions [33]. The International Union for Conservation of Nature (IUCN) Red List of Threatened Species provides a standardised and comprehensive assessment of global extinction risk for thousands of species [34]. Its popularity amongst zoos and wildlife managers has resulted in its categorical designations being routinely used in studies assessing the prioritisation of species for conservation initiatives as well as those evaluating zoo collection composition [29,31,35]. However, using global threat status in isolation can overlook important regional and local conservation needs — for example, species that are globally secure may be scarce or declining within particular countries — potentially leading to inefficient allocation of time and resources [16]. To capture these finer-scale patterns, many countries and subnational jurisdictions maintain their own threatened-species lists, which help identify at-risk species, inform policy and legislation, and guide national conservation priorities [36]. Jurisdictions may also prioritise species for cultural, spiritual or other reasons that contribute to human wellbeing [37]. In addition, peripheral populations often represent genetically distinct populations that warrant targeted conservation attention [38]. For these reasons, some contemporary zoos and conservation organisations have developed breeding programmes for locally threatened species [39], but exactly what proportion of a zoos collection is used for this purpose is widely understudied. In the UK, the British & Irish Association of Zoos & Aquariums (BIAZA) suggest that all animals housed in collections should be ascribed a role; for example, exhibit or education value, *ex situ* or conservation breeding or research [22]. One example of a species that is held in high numbers in the UK for its national conservation value is the European wild cat (*Felis silvestris).* Although categorised as Least Concern on the IUCN Red List, its populations have declined drastically in the UK through persecution and hybridisation, despite having a stable population across Europe [40].

The UK has several species that are of global Least Concern, however, find themselves onto local priority lists for conservation, such as the European water vole (*Arvicola amphibius),* European pine martin (*Martes martes)* and the European polecat *(Mustela putorius).* Priority species are identified as having a significant ecological or conservation value. Understanding numbers and locations of captive threatened species can aid the management of breeding programmes and improve genetic diversity, increasing their chances of survival in the wild [33]. Several studies have examined the proportion of threatened species held in zoos globally [1,21,32]; however, there have been no published attempts to quantify the numbers found in a regional association such as BIAZA that hold their own motivations for collection planning, conservation and education. Regional associations manage cooperative *ex situ* population management and breeding programmes like the Association of Zoos and Aquariums Species Survival Plan (SSP) [41] or the EAZA *Ex situ* Programme (EEP). These programmes maintain healthy, genetically diverse and demographically stable populations of threatened species to support wild populations or for reintroduction to the wild [42]. In addition to its recognised native species, the UK designates species that are regionally threatened or of conservation concern through formal priority lists. Determining the captive diversity of these species can better aid the decision-making process of zoo and wildlife managers, safeguarding the future of the most regionally threatened species and strengthening the adoption of a *One Plan Approach*.

This study focuses on vertebrates within BIAZA collections and has three primary aims: (i) to quantify population size and species diversity across vertebrate classes; (ii) to compare the proportions of globally threatened and non-threatened species; and (iii) to evaluate the representation of UK native and priority species. This research will help to demonstrate the motivations of a contemporary zoos collection planning. Having knowledge of the diversity of BIAZA’s threatened species, particularly for those that feature on lists of regional priority will be key for the success of *ex situ* conservation breeding programmes, as well as providing a captive sanctuary prior to the possibility of reintroduction or release.

## Materials and methods

### Species 360

Species 360 is a worldwide zoological information system (often referred to as ZIMS) that maintains an online database of captive species. The platform is designed to manage animal accessions and dispositions, behavioural observations, feed logs and other relevant husbandry practices (ZIMS 2024). A ZIMS licence was obtained for June 2023 – May 2024 that allowed for access to *ex situ* populations which were filtered to include BIAZA members only. A total of 106 BIAZA members were found to be using the data management system, 86% of BIAZA’s membership. A further 7 stocklists were acquired via a Freedom of Information act 2000 (FOI) request resulting in a total sample size of 113 organisations, 92% of all BIAZA members. The 8% of BIAZA members (10 institutions) not recorded failed to respond to FOI requests and constituted 7 small and 3 medium sized institutions as per the standards of modern zoo practice for Great Britain [43]. All vertebrate species were extracted from ZIMS members and FOI stocklists and sorted into the five major classes (fish, amphibians, reptiles, birds and mammals). Each class was further organised into family, genus and species, along with the total number of captive individuals and associated IUCN Red List classification. Invertebrate species were excluded from the analysis due to their categorisation into groups and colonies making data analysis incomparable with vertebrate species.

### Globally threatened species

To establish the total number of globally threatened vertebrate species in BIAZA collections, all species were cross referenced with the IUCN Red List (https://www.iucnredlist.org) and organised into their respective threat categories.

To determine the number of globally threatened species that inhabit the UK, lists were retrieved using the advanced search option on the IUCN Red List of Threatened Species website. Specifically, species were filtered for threat class (Threatened: Critically Endangered (CR), Endangered (EN), and Vulnerable (VU); Non-threatened: Near Threatened (NT), and Least Concern (LC)), land region (UK), and taxonomy (Mammals, Birds, Fish, Reptiles, Amphibians). The list was further supplemented with migratory oceanic species whose ranges intersected with the British Isles Exclusive Economic Zone (EEZ) in ArcGIS 10.5. The other IUCN categories referenced in this study but not included in the analysis of threatened vs non-threatened species include Extinct in the Wild (EW), Extinct (EX), Data Deficient (DD), Not Evaluated (NE), and Conservation Dependent (CD). A further 57 species (n = 2,180 individuals) were classified to the level of genus due to a lack of taxonomic data at the species level. In addition, 60 domesticated breeds (n = 1,198 individuals) were identified but not included in the analysis due to a lack of Red List categorisation. Both groups were included in the data set to provide accurate total numbers of species and individuals, however removed from the analysis related to IUCN Red listings due to a lack of information.

### Regionally threatened species

To determine the number of British native vertebrate species present in BIAZA collections, a full list was compiled from the Mammal Society’s British mammal list [44]; British Ornithologists’ Union British List [45]; British Herpetological Society native reptile and amphibian list [46]; and Fishbase’s native British species list [47].

To ascertain the number of statutory priority species, lists were obtained from Lim, Starnes [48] supplementary dataset. Specifically, the UK priority species list is a compilation of lists from the four UK countries. Data was obtained from the Section 41 species list for England [49], the Northern Ireland priority lists [50], the Scottish priority list [51], and the Biodiversity Information Service for Powys and the Brecon Beacons National Park (BBNP) that supplemented Section 7 priority species for Wales [52].

### Data analysis

The Shannon Diversity Index$\;(H^{\prime }=-\sum \;(\rho \text{i}*\;I\text{n}(\rho \text{i})$) was used because it incorporates both species richness and the relative abundance of each species. As the primary goal of *ex situ* populations is to preserve genetic diversity – often by safeguarding rare or low-abundance species – the Shannon Diversity Index’s responsiveness to changes in these less common species makes it an appropriate metric for tracking their status [53].

Kruskal- Wallis tests were used to determine whether there were significant differences between the population sizes of species across vertebrate class due to observed unequal variances (Lavene’s test *p* < 0.001). A Dunns post hoc test with Holm corrections was used to determine differences between classes. Due to the non-normality of the data, a Mann-Whitney test was used to determine whether there were significant differences between the number of individuals in threatened (CR, EN, VU,) versus non threatened (NT, LC) categories (Shapiro-wilk *p* < 0.001). Further Kruskal-Wallis tests were used to ascertain whether there were significant differences between the numbers of individuals in threatened categories across vertebrate class due to observed unequal variances (Lavene’s test *p* < 0.001). A Dunns post hoc test with Holm corrections showed which groups varied significantly from each other. ANOVA testing was used to determine whether there were significant differences between the population sizes of native species across vertebrate class due to observed equal variances (Lavene’s test *p* < 0.090). The normality assumption was accepted specifically for the native species subset. A final Kruskal-Wallis was test was used to establish whether there were significant differences between the number of priority UK species in each class (Lavene’s test *p* < 0.001).

## Results

### Population size and species diversity

**Fig 1:**
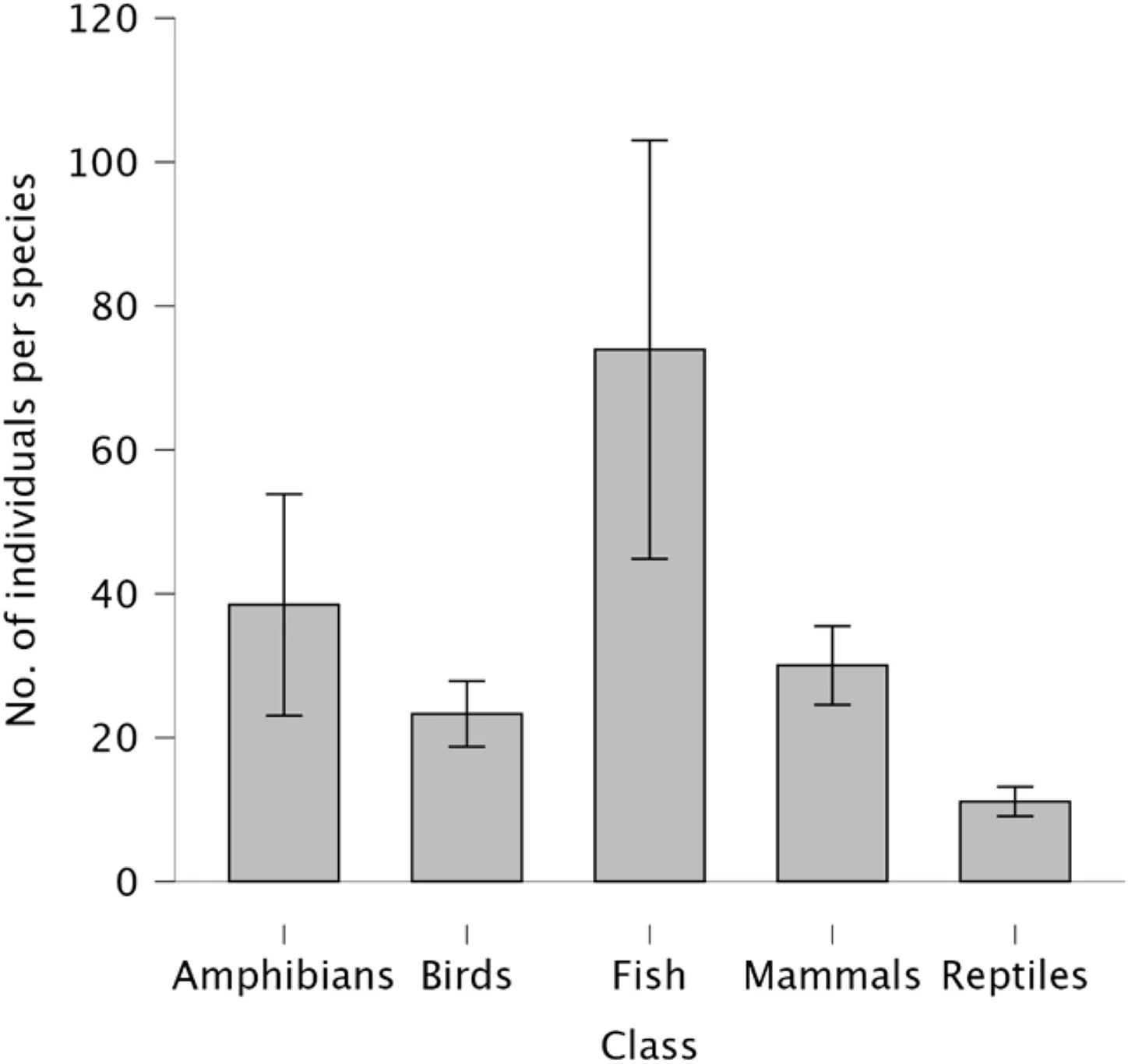
Mean population size of each captive species across vertebrate classes within BIAZA institutions (with associated upper and lower 95% confidence intervals).

The list of vertebrate taxa compiled for BIAZA institutions included 106,240 organisms across 2,629 species and five vertebrate classes (number of individuals per species: min = 1, max = 8827, average = 40, SD = 257) (Table 1). Fish made up the greatest proportion of captive individuals with 61% of the collections total (birds 16%, mammals 15%, amphibians 5%, reptiles 4%). Fish also displayed the highest mean number of individuals per species (Fig 1) and highest species richness. Reptiles made up only 4% of BIAZA stocklists, with the smallest number of individuals (n = 4,269) and lowest mean of individuals per species (Fig 1). Birds exhibited the highest diversity in collections (*H’* = 5.397), whereas amphibians showed the lowest diversity (*H’* = 3.716) and the smallest number of species (sp. 130). High (H’) values were noted for all five vertebrate classes indicating a high diversity amongst BIAZA collections (Table 1).

**Table 1:** Number of species (*Sp.*) and individuals (*No.*) for each vertebrate class found in BIAZA collections per IUCN Red List category.

Class/IUCN	LC	NT	VU	EN	CR	EW	EX	DD	NE	CD	Total	Percentage Threatened	Diversity Score (H)
**Mammals *Sp.***	238	41	64	67	33	0	0	25	0	0	529	31%	5.228
**Mammals *No.***	8,505	1,040	1,229	1,983	1,033	0	0	909	0	0	15,898	27%	
**Birds *Sp.***	511	72	61	43	23	1	0	0	0	0	715	17%	5.397
**Birds *No.***	10,782	2,151	1,834	1,221	646	19	0	0	0	0	16,669	22%	
**Reptiles *Sp.***	224	22	46	39	31	0	0	7	6	2	384	30%	5.072
**Reptiles *No.***	2,211	276	528	443	588	0	0	24	164	4	4,269	37%	
**Amphibians *Sp.***	75	12	14	11	11	0	0	3	0	0	130	27%	3.716
**Amphibians *No.***	2,176	221	511	860	1,104	0	0	91	0	0	5,001	49%	
**Fish *Sp.***	545	27	40	39	26	5	0	38	107	0	871	12%	4.489
**Fish *No.***	33,957	900	2,919	4,292	11,031	1,547	0	1,043	6,584	0	64,403	28%	

Percentages indicate the proportion of threatened individuals and species within each class. IUCN categories: least concern (**LC**), near threatened (**NT**), vulnerable (**VU**), endangered (**EN**), critically endangered (**CR**), extinct in the wild (**EW**), extinct (**EX**), Data Deficient (**DD**), not evaluated (**NE**), conservation dependent (**CD**). Columns highlighted in grey indicate threatened categories.

Species abundance was found to vary significantly with vertebrate class (Kruskal Wallis: H = 65.96, *p* < 0.001). Dunn’s post hoc test with Holm corrections indicated that reptiles (median = 4) scored significantly lower mean numbers of individuals per species than amphibians [9], mammals [9], birds [7] and fish [7] (all *p* < 0.001, *r*_*rb*_ = 0.184–0.313). Amphibians and mammals also displayed higher scores than birds (*p* = 0.081, *r*_*rb*_ = 0.136 and (*p* < 0.001, *r*_*rb*_ = 0.112 respectively). All other comparisons were non-significant.

### IUCN threatened species

**Fig 2:**
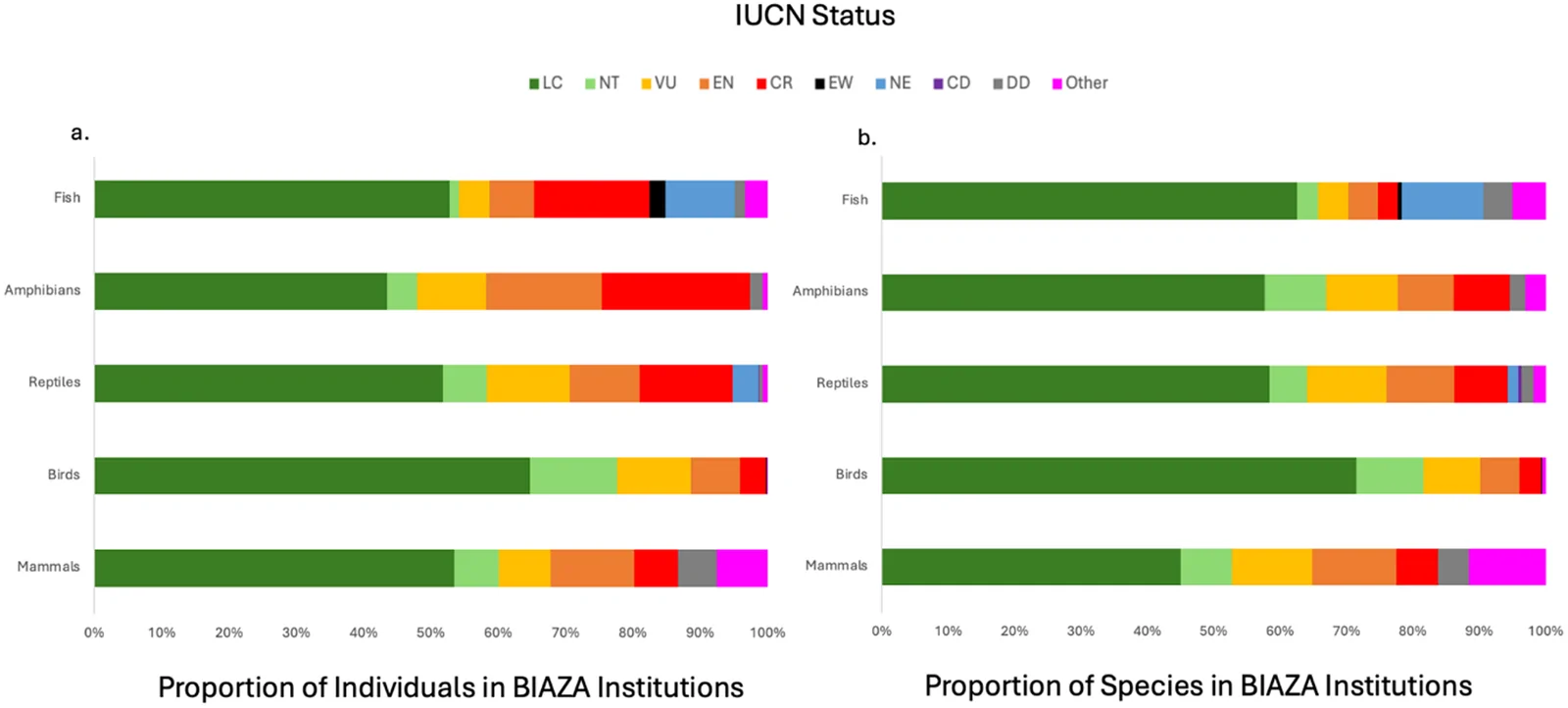
Proportion of BIAZA’s total number of individual organisms (a) and species (b) holdings in each IUCN category. Threatened categories: vulnerable (**VU**), endangered (**EN**), critically endangered (**CR**); non-threatened: near threatened (**NT**), least concern (**LC**); additional IUCN categories: extinct in the wild (**EW**), not evaluated (**NE**), conservation dependent (**CD**), data deficient (**DD**); Other: domesticated and those categorised to the level of genus only.

A total of 30,222 vertebrate individuals within assessed BIAZA collections fall into threatened categories (VU, EN, CR) of the IUCN Red List, 28.5% of the total amount of captive individuals (Table 1; Fig 2). A further 62,219 individuals were found to be in non-threatened categories, 58% of BIAZA’s total captive population. The remaining 13.5% (n = 13,799) excluded from the analysis consist of domesticated breeds (n = 1,198), species classified as EW (n = 1,566), DD (n = 2,067), NE (n = 6,748) or CD (n = 4), and those individuals categorised to the level of genus (n = 2,216). The results of the Mann-Whitney test indicated that threatened species had a significantly higher mean number of individuals per species than non-threatened species (*U* = 412,043.5, *p* < 0.001).

Fish contained the greatest abundance of individuals in threatened categories (n = 18,242) with over 60% of these individuals found in the highest threat category (CR). Reptiles had the lowest abundance of threatened individuals (n = 1,559), although due to the smaller captive population, their proportional representation (37%) amongst threatened categories remains higher than birds, fish, and mammals. Amphibians have 2,475 threatened individuals. Due to the smaller total population, amphibians have the largest proportional representation (49%) in threatened categories. Amphibians display a near equal proportion of threatened and non-threatened (48%) individuals (Fig 2a). Captive bird populations display the lowest proportional representation (22%) of individuals in threatened categories of any observed class. Additionally, six non-native species classified as EW were identified, namely the Golden Skiffia (*Skiffia francesae*), Monterrey Platyfish (*Xiphophorus couchianus*), Potosi Pupfish (*Cyprinodon alvarezi*), La Palma Pupfish (*Cyprinodon longidorsalis*), Charco Palma Pupfish (*Cyprinodon veronicae*) and Socorro Dove (*Zenaida graysoni*).

Results from the Kruskal-Wallis test indicated a significant effect of class on threatened species abundance (*H* = 22.03, *p*= < 0.001). Amphibians exhibited the highest median [19], followed by fish [12], mammals [11], birds [9] and reptiles [6]. Dunn’s post hoc test with holm corrections indicated that reptiles had significantly lower numbers than amphibians (*p* = 0.005, *r*_*rb*_ = 0.38), fish (*p* = 0.001, *r*_*rb*_ = 0.27), and mammals (*p* = 0.019, *r*_*rb*_ = 0.23). All other comparisons were non-significant. Results indicate that the overall effect is driven by lower scores for reptiles relative to amphibians, fish and mammals.

### UK species

**Fig 3:**
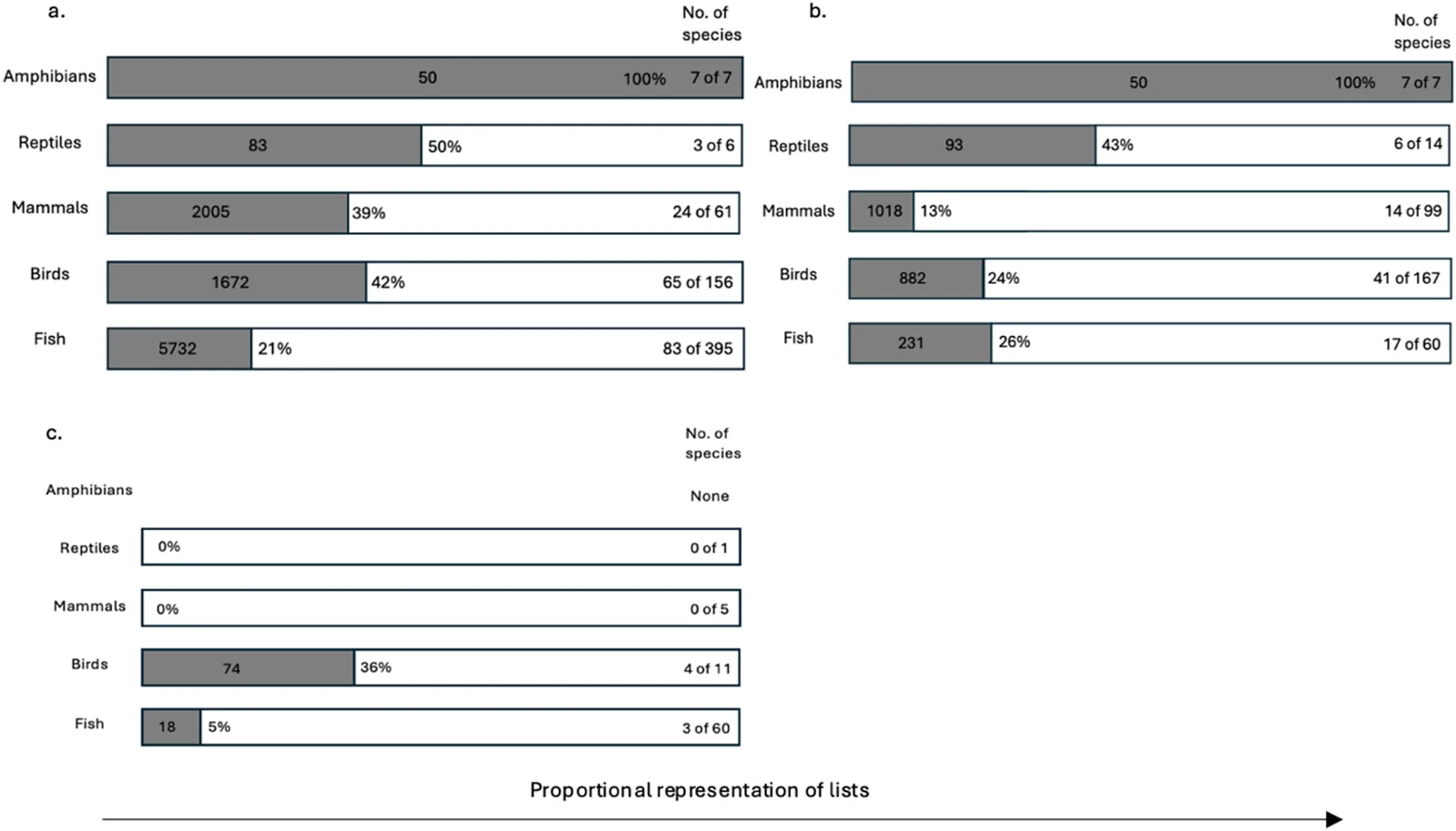
Number and proportion (%) of species found on UK native (a), priority (b), and globally threatened (c) lists. The total number of species that make up each list are provided at the end of each row.

The list of UK native vertebrate taxa in BIAZA collections totalled 9,548 organisms across 182 species within the five vertebrate classes (number of individuals per species: min = 1, max = 1957, average = 52.5, SD = 170.5) (Fig 3a). The UK’s lists of native and priority amphibians comprise the same seven species, all of which are found in BIAZA collections – Common Frog (*Rana temporaria*), Pool Frog (*Pelophylax lessonae*), Natterjack Toad (*Epidalea calamita)*, Common Toad (*Bufo bufo*), Great Crested Newt (*Triturus cristatus*), Smooth Newt (*Lissotriton vulgaris*) and Palmate newt (*Lissotriton helveticus*). Fish display the lowest proportional representation (21%) holding only 83 of the listed 395 native species. However, when observing individual numbers of native species, amphibians (n = 50) and reptiles (n = 83) have comparatively lower population sizes to birds (n = 1,672), mammals (n = 2,005) and fish (n = 5,732). Abundance of native species did not vary significantly across class (ANOVA: *F*(4, 177) = 0.93, *p* = 0.45).

The list of UK priority taxa totalled 2,274 organisms across 84 species (number of individuals per species: min = 1, max = 514, average = 27.1, SD = 62.7) (Fig 3b). Mammals are the most abundant class (n = 1,018) although their proportional representation on priority lists was the lowest of any class (13%). There were no significant differences in the abundance of priority species between classes (Kruskal-Wallis: *H* = 5.35, *p* = 0.25).

The list of globally threatened taxa native to the UK totalled 92 individuals across fish and bird classes (number of individuals per species: Min = 1, Max = 37, average = 13, SD = 12.1) (Fig 3c). These species included the Grey Triggerfish (*Balistes capriscus*), Atlantic Cod (*Gadus morhua*), Atlantic Horse Mackerel (*Trachurus trachurus*), Common Pochard (*Aythya ferina*), Snowy Owl (*Bubo scandiacus*), Long-tailed Duck (*Clangula hyemalis*) and European Turtle Dove (*Streptopelia turtur*). There are no globally threatened mammal, reptile or amphibian species native to the UK in assessed BIAZA collections. Birds have the greatest abundance of globally threatened species native to the UK (n = 74) and the greatest proportional representation (36%).

## Discussion

This research is the first to document the diversity and abundance of vertebrate species in BIAZA collections and has hlped provide an accurate account of the current state of play in British zoological institutions. The examination of stocklists highlights BIAZA’s diverse collections across all five vertebrate classes. The high Shannon diversity values for each class indicate high species richness and evenness across BIAZA’s captive populations when compared with other research [54–57]. Akinboboye, Adetola [54] used the Shannon index to assess the diversity of whole zoo collections with values ranging from H = 3.75 to H = 1.56. Shannons index has been used in other *ex situ* examples including the diversity of individual aquarium exhibits [55], avian diversity in ZIMS data [56], and the diversity of *Agamidae* species in global zoological institutions [57]. This research is the first of its kind to provide a diversity score for an associations collection, providing a reference point for other regional comparisons. Research into historic and current trends for zoo collection planning has predominantly examined the number of species in global collections [1,21,58]. However, the additional examination of the number of individuals per species provides a more comprehensive and accurate understanding of the diversity amongst classes, serving as an important tool for the management of breeding programmes, studbooks, and transfers of organisms between institutions [32].

There is considerable research that suggests a public preference towards mammals [22,29,31]. Similarly, research into captive breeding programmes [14] and research and research training [6] show mammals, particularly from the orders *Primate* and *Carnivora* to be the most popular. Despite this public preference for mammals, both this investigation and historic research into collection planning show birds and fish to be the best represented in terms of species prevalence [21,58]. The greater number of fish and bird species may in part be attributed to the nature in which they are held captive. Birds and fish are kept in three-dimensional enclosures, maximising use of available space. Both classes are often kept in mixed species enclosures making it comparatively easier for institutions to stock a greater number of species whilst using space efficiently [59]. Furthermore, several studies have suggested that the social behaviours of flocking in birds [60] and shoaling in fish [61] can influence zoo and aquarium collection planning through their demonstrated effects on welfare and reproduction. These effects are formalised into husbandry guidelines and then operationalised into collection plans and species selection decisions [62].

The relative scarcity of amphibians amongst BIAZA institutions is of concern and mirrors global trends [21,63,64]. As most amphibians are comparatively small and less active than other vertebrate taxa, they are often disregarded as a charismatic species [65]. Research also suggests that the high sensitivity of amphibians to water quality, temperature and disease often requires specialised and expensive husbandry infrastructure, making amphibians (and to a lesser extent some reptiles) more expensive per unit exhibit [66]. Public attitude studies underline the perception of low charisma for amphibians, with widespread expressions of disgust and negative folklore reported across cultures [67,68]. It is perhaps for this reason that populations sizes of amphibians have remained low as their ability to attract the public remains questioned. Amphibians are currently one of the most threatened taxa due to their sensitivity to disease, habitat loss, pollution and climate change [69]. Their presence in zoo collections can therefore play a crucial role in conservation management, utilising their threat status to better inform the public of their ecological significance and risk of extinction [70]. Although numbers of amphibians are low, it is encouraging to see 49% of BIAZA’s total abundance are categorised as threatened with 22% of those falling into CR designation. However, the high representation of threatened amphibians may partly result from the rapid deterioration of amphibian conservation status globally [27]. Many species held in captivity may not have been threatened when acquired but subsequently became threatened as wild populations declined.

The absence of globally threatened mammals and reptiles native to the UK is likely attributed to the pelagic and migratory nature of those organisms. The Leatherback Turtle (*Dermochelys coriacea*) for example is the only reptile present on UK threatened lists however, due to its behavioural ecology the species is not kept in captivity. Similarly, only Cetacea make up the total list of threatened mammals including the Sperm whale (*Physeter macrocephalus*), Fin Whale (Balaenoptera physalus), Blue whale (*Balaenoptera musculus*) and Sei Whale (*Balaenoptera borealis).*

It is estimated that 21% of all reptile species worldwide are threatened [71], and despite having a greater proportional representation on native and priority lists than mammals, birds and fish, reptiles are historically under-resourced in their conservation management and research [72]. Although results show reptiles to be the least abundant class, the high (H’) value (5.082) suggests more consistent population sizes per species compared with other classes. The abundance of reptiles held in UK zoos increased by 47% between 2003 and 2023, with specific increases noted in the orders *Testudines* (40%), *Lacertilia* (102%), and *Crocodylia* (147%) [73]. The large increase in *Crocodylia* appears driven by a favour to house large, visually impressive (or charismatic) species that attract visitors. The increase in crocodilian abundance is largely due to increased holdings of the Nile Crocodile *(Crocodylus niloticus) (*n = 37*)* and Siamese crocodile *(Crocodylus siamensis)* (n = 21), the two most abundant crocodilians in BIAZA collections. This increase in reptile holdings may account for the high diversity recorded amongst BIAZA collections. The smaller and more consistent populations sizes noted with reptiles maybe a consequence of zoos attempts to keep smaller and more sustainable populations sizes [74]. Large population sizes are often not required to communicate an effective conservation message with reptiles often housed in smaller, single species exhibits.

The total abundance of UK native (9%) and priority (2%) species as a proportion of BIAZA’s absolute numbers is comparatively low to that of globally threatened categorisations (28.5%). A similar trend in collections was noted by Kerr, Sauve [32], who found North American zoos hold locally threatened species slightly less often than would be expected by chance. Therefore, the direct contribution of *ex situ* populations to locally threatened species remains limited in terms of taxonomic coverage. The importance of maintaining local priority species that may not be considered threatened elsewhere, is vital for populations at the edge of a species geographic range. An example of this is with *F. silvestris.* BIAZA’s captive population of 69 individuals is significant considering the UK’s last wild populations are estimated to only number a few hundred [75], a vital insurance policy for the future survival of the species. Similarly, BIAZA contains sizeable populations of the European Water Vole (*A. amphibius*) which is EN on the Red List for British mammals, and the European Pine Martin *(M.martes*) which is classified as CR in England and Wales, however both remain classified as LC on the global IUCN Red list. These *ex-situ* populations have helped contribute to successful, collaborative, reintroduction programmes throughout the UK. For example, Chester Zoo has been a major contributor to the Pine Marten recovery project, restoring viable, self-sustaining populations of *M. martes* to England and Wales [76]. Similarly, The Wildfowl and Wetland Trust (WWT) working in partnership with local wildlife trusts and landowners have reintroduced *A. amphbius* at several wetland sites including Llaneli, Slimbridge and Martine Mere [77]. Successful BIAZA reintroduction programmes are evident for species in other classes including the: Agile Frog (*Rana dalmatina*), Red Billed Chough (*Pyrrhocorax pyrrhocorax*) and White Clawed Crayfish (*Austroptamobius pallipes*) [78].

BIAZA’s adoption of collaborative conservation is helping with the survival of several species categorised as Extinct in the Wild under the IUCN Red List. For example, The Zoological Society of London (ZSL) are part of an international effort to breed and reintroduce the Socorro Dove (*Zenaida graysoni*) back to its native island off the west coast of Mexico [79]. These examples highlight the importance of combining stakeholder resources to produce one comprehensive conservation strategy for both global and regionally targeted species.

*Ex situ* data management tools like ZIMS can assist BIAZA members with breeding at British and Irish (B&I) and European (EEP) scales and the management of European Studbooks (ESB) through accurate cataloguing of species births, deaths and transfers. The recent integration of ZIMS *ex situ* population data with the IUCN Red List provides a complete image of species populations numbers in the wild and human care [80]. This research would encourage all BIAZA members to integrate their *ex situ* populations with data management systems allowing for better sharing of taxonomic data and a significant advancement in a *One Plan Approach*.

It must be noted that the number of species and their population sizes is only one measure of an institutions collection planning. This approach does not account for the resources, time, space, research output and marketing required for each species to be housed [81]. Further research into these variables may provide more robust evidence as to why reptile numbers differ significantly from other vertebrate taxa.

## Conclusion

Given the fragility of wild habitats and their constant battle for space with human population and development, species at every trophic level of terrestrial and aquatic ecosystems find themselves at risk [82,83]. Captive populations will therefore play an increasingly important role in the conservation of the most threatened species, providing a refuge from persecution or a temporary base before the possibility of reintroduction. This research has demonstrated the high levels of diversity across vertebrate classes as BIAZA institutions display a wide range of animals across all taxa. This preference to favour a high diversity of species over an attempt to house only threatened species helps to highlight the motivations of an institution in addition to the wider association. A diverse collection of animals can serve as an effective pedagogical tool for institutions and even those species not categorised as threatened can contribute to a narrative of habitat loss and fragmentation [74]. Previous studies looking into zoological collections are limited by their observations of species number alone [1,21,58]. The addition of captive population numbers provides a more accurate assessment of their capability to contribute to both *ex* and *in situ* conservation practices.

Whilst BIAZA contribute to housing both native and local priority species, the practice is evident for only a small number of species. As conservation is a prerequisite of BIAZA membership, more could be done to house a greater proportion of locally threatened species as well as holding them in greater population sizes. Measuring the financial and time burden of keeping certain species together with geographical complexities surrounding zoo location relative to that of wild populations remains largely undetermined. The preservation of local priority and threatened species can be valuable for maintaining populations at the edge of a given species range. The European wildcat for example, highlights the importance of maintaining a captive population as wild numbers in Britain decline. This example highlights the importance of maintaining both global and regional lists as there use in isolation may result in the misallocation or ineffective use of resources. Zoo and aquarium facilities provide an important space for human and animal interaction. These interactions help to stimulate public interest in the natural world and increase visitor awareness of threatened species and habitat loss. With a global trend of people moving to urban areas, zoos find themselves ideally positioned to influence the mindset of a growing visitor pool on environmental best practise. This responsibility will be key for the future safeguarding of our wild spaces and most threatened species.

## Supporting information

S1 File BIAZA vertebrate species and UK native species list. (XLSX)
