# Supplementary material for: Taking stock: Can captive species contribute to biodiversity conservation?
Source: PLoS One. 2026 Aug 19;21(8):e0354602. doi: 10.1371/journal.pone.0354602 (PMC13489450; doi:10.1371/journal.pone.0354602)
Supplement: Supplementary file 1 [file pone.0354602.s002.docx]

Dear Reviewers,

Firstly, thank you for taking the time to read my paper and providing me with your comments and suggestions. I have addressed all reviewer recommendations and below, provide detail of the actions taken.

- Reviewer 1, comment 1- “A distinction might be useful here. Housing threatened species only makes conservation sense if they are used for research or breeding and release that benefits wild populations. Holding them alone does not mean any conservation action is actually taking place. And in cases where the ex-situ populations of animals are not sustainably managed, it may actually be more ethical to house non-threatened species, since removal from the wild is less likely to impact species’ status”.
- Response (line 86-90) – Clarification provided over the holding of threatened species and their contribution to conservation. Three new references provided (23-25).
- Reviewer 1, comment 2 “I don’t dispute that covering 92% of BIAZA members should provide a good overview of the status of BIAZA collections. However, did the authors identify any biases in the institutions they weren’t able to access? For instance, if that 8% covered mostly small, regional zoos, then they might be missing out on collections of regionally important species. Alternatively, if it was a random sampling that were missed, then it would be less likely to introduce biases. This extra information would be helpful for readers to assess the quality of the sample”.
- Response (line 163-165) – Provided the number of institutions missing from the analysis along with size of institution. However, to protect Institutional anonymity and consistency with the rest of the document, no names of intuitions are provided. Reference 43 added.
- Reviewer 1, comment 3 - “Table 1: the figure capture is missing a description for “DD”, which presumably is data deficient.”
- Response (line 250) – Added missing Data Deficient (DD) criterion to table 1 legend.
- Reviewer 1, comment 4 - “The cause and effect here is hard to disentangle. Yes, a high proportion of BIAZA’s amphibian species are threatened, but might that not simple be because amphibians are more threatened globally than other species (line 89)? It does not mean these species were selected for collection planning because of their status but could be that wild population trends have been so poor that more of the species held have become threatened over time”.
- Response (378-381) – Provided clarifying sentence around the reason for high number of threatened amphibians with the provision of reference 27.
- Reviewer 2, comment 1 In lines 222-224, the authors state they used an ANOVA test to analyse native species population sizes across vertebrate classes because homoscedasticity was met (Lavene's test p<0.090). However, since the overarching dataset presented non-normal distribution patterns (as stated for the Mann-Whitney and Kruskal-Wallis segments), please clarify if the normality assumption was verified specifically for the native species subset to validate the use of a parametric test.
- Response (line 224-225) – Added line stating the use of parametric testing specifically for native species subset.
- During the resubmission a small error was noticed with figure 3c. Author changed fish representation from 6 species to 3 as 3 of the species were categorised as DD rather than a threatened categorisation. The proportional representation of fish was changed from 10% to 5% and the number of individuals from 39 to 18. On line 326-327 The necessary changes were made to the MIN, MAX, AVERAGE and SD for the number of individuals per species for globally threatened species native to the UK. This minor edit had no change to the statistical analysis or discussion

Thank you once more for the comments regarding this paper and I look forward to finding out the revised outcome.

Kind regards,

Justin Lennon

PhD student
