# Supplementary material for: Taking stock: Can captive species contribute to biodiversity conservation?
Source: PLoS One. 2026 Aug 19;21(8):e0354602. doi: 10.1371/journal.pone.0354602 (PMC13489450; doi:10.1371/journal.pone.0354602)
Supplement: Peer review history

# Peer review: Decision Letter 0

**DOI:** 10.1371/journal.pone.0354602.r001

**Authors:** Ulrike Gertrud Munderloh

19 May 2026

PONE-D-26-02051

Taking Stock: Can captive species contribute to biodiversity conservation?

PLOS One

Dear Dr. Lennon,

Thank you for submitting your manuscript to PLOS ONE. After careful consideration, we feel that it has merit but does not fully meet PLOS ONE’s publication criteria as it currently stands. Therefore, we invite you to submit a revised version of the manuscript that addresses the points raised during the review process.

Both reviewers agree that your study is valuable and robust. However, they also raise important though minor concerns that must be addressed concerning the validity of keeping captive species at zoos, and data analysis.

Please submit your revised manuscript by Jul 03 2026 11:59PM. If you will need more time than this to complete your revisions, please reply to this message or contact the journal office at plosone@plos.org. When you're ready to submit your revision, log on to https://www.editorialmanager.com/pone/ and select the 'Submissions Needing Revision' folder to locate your manuscript file.

Please include the following items when submitting your revised manuscript:

- A letter that responds to each point raised by the academic editor and reviewer(s). You should upload this letter as a separate file labeled 'Response to Reviewers'.
- A marked-up copy of your manuscript that highlights changes made to the original version. You should upload this as a separate file labeled 'Revised Manuscript with Track Changes'.
- An unmarked version of your revised paper without tracked changes. You should upload this as a separate file labeled 'Manuscript'.

If you would like to make changes to your financial disclosure, please include your updated statement in your cover letter. Guidelines for resubmitting your figure files are available below the reviewer comments at the end of this letter.

If applicable, we recommend that you deposit your laboratory protocols in protocols.io to enhance the reproducibility of your results. Protocols.io assigns your protocol its own identifier (DOI) so that it can be cited independently in the future. For instructions see: https://journals.plos.org/plosone/s/submission-guidelines#loc-laboratory-protocols. Additionally, PLOS ONE offers an option for publishing peer-reviewed Lab Protocol articles, which describe protocols hosted on protocols.io. Read more information on sharing protocols at https://plos.org/protocols?utm_medium=editorial-email&utm_source=authorletters&utm_campaign=protocols.

As the corresponding author, your ORCID iD is verified in the submission system and will appear in the published article. PLOS supports the use of ORCID, and we encourage all coauthors to register for an ORCID iD and use it as well. Please encourage your coauthors to verify their ORCID iD within the submission system before final acceptance, as unverified ORCID iDs will not appear in the published article. *Only*  the individual author can complete the verification step; PLOS staff *cannot*  verify ORCID iDs on behalf of authors.

We look forward to receiving your revised manuscript.

Kind regards,

Ulrike Gertrud Munderloh, Ph.D.

Academic Editor

PLOS One

Journal Requirements:

When submitting your revision, we need you to address these additional requirements.

1. Please ensure that your manuscript meets PLOS ONE's style requirements, including those for file naming. The PLOS ONE style templates can be found at

https://journals.plos.org/plosone/s/file?id=wjVg/PLOSOne_formatting_sample_main_body.pdf and

https://journals.plos.org/plosone/s/file?id=ba62/PLOSOne_formatting_sample_title_authors_affiliations.pdf.

2. If the reviewer comments include a recommendation to cite specific previously published works, please review and evaluate these publications to determine whether they are relevant and should be cited. There is no requirement to cite these works unless the editor has indicated otherwise.

3. Please review your reference list to ensure that it is complete and correct. If you have cited papers that have been retracted, please include the rationale for doing so in the manuscript text, or remove these references and replace them with relevant current references. Any changes to the reference list should be mentioned in the rebuttal letter that accompanies your revised manuscript. If you need to cite a retracted article, indicate the article’s retracted status in the References list and also include a citation and full reference for the retraction notice.

[Note: HTML markup is below. Please do not edit.]

Reviewers' comments:

Reviewer's Responses to Questions

**Comments to the Author**

1. Is the manuscript technically sound, and do the data support the conclusions?

The manuscript must describe a technically sound piece of scientific research with data that supports the conclusions. Experiments must have been conducted rigorously, with appropriate controls, replication, and sample sizes. The conclusions must be drawn appropriately based on the data presented.

Reviewer #1: Yes

Reviewer #2: Yes

**********

2. Has the statistical analysis been performed appropriately and rigorously?

Reviewer #1: Yes

Reviewer #2: Yes

**********

3. Have the authors made all data underlying the findings in their manuscript fully available?

The PLOS Data policy requires authors to make all data underlying the findings described in their manuscript fully available without restriction, with rare exception (please refer to the Data Availability Statement in the manuscript PDF file). The data should be provided as part of the manuscript or its supporting information, or deposited to a public repository. For example, in addition to summary statistics, the data points behind means, medians and variance measures should be available. If there are restrictions on publicly sharing data—e.g. participant privacy or use of data from a third party—those must be specified.

Reviewer #1: Yes

Reviewer #2: Yes

**********

4. Is the manuscript presented in an intelligible fashion and written in standard English?

PLOS ONE does not copyedit accepted manuscripts, so the language in submitted articles must be clear, correct, and unambiguous. Any typographical or grammatical errors should be corrected at revision, so please note any specific errors here.

Reviewer #1: Yes

Reviewer #2: Yes

**********

5. Review Comments to the Author

Please use the space provided to explain your answers to the questions above. You may also include additional comments for the author, including concerns about dual publication, research ethics, or publication ethics. (Please upload your review as an attachment if it exceeds 20,000 characters)

Reviewer #1: As zoological institutions continue to brand themselves as conservation organizations, I think it is a valid exercise to investigate the actual conservation value that their collections provide. Therefore I read this article with great interest. Overall the paper is clearly written, well supported by references, and its conclusions appear supported by the data they share.

It comes across as an assumption in multiple places throughout the paper (e.g. title, intro, discussion) that holding threatened/endangered species counts as helping conserve them, regardless of the other actions taken. While they do highlight some nice case studies in the UK where further conservation action is taken (e.g. reintroductions), this is not the case for the majority of threatened species held ex-situ in BIAZA institutions. It would be helpful to clarify that the contributions of zoological institutions to conservation requires more than just holding species, but actively breeding, releasing or mounting public support for conservation action, etc.

I have a few minor points of clarification that would be helpful for the authors to consider, as detailed below:

Line 86-87 A distinction might be useful here. Housing threatened species only makes conservation sense if they are used for research or breeding and release that benefits wild populations. Holding them alone does not mean any conservation action is actually taking place. And in cases where the ex-situ populations of animals are not sustainably managed, it may actually be more ethical to house non-threatened species, since removal from the wild is less likely to impact species’ status.

-I don’t dispute that covering 92% of BIAZA members should provide a good overview of the status of BIAZA collections. However, did the authors identify any biases in the institutions they weren’t able to access? For instance, if that 8% covered mostly small, regional zoos, then they might be missing out on collections of regionally important species. Alternatively if it was a random sampling that were missed, then it would be less likely to introduce biases. This extra information would be helpful for readers to assess the quality of the sample.

Table 1: the figure capture is missing a description for “DD”, which presumably is data deficient.

Line 373- The cause and effect here is hard to disentangle. Yes, a high proportion of BIAZA’s amphibian species are threatened, but might that not simple be because amphibians are more threatened globally than other species (line 89)? It does not mean these species were selected for collection planning because of their status, but could be that wild population trends have been so poor that more of the species held have become threatened over time.

Reviewer #2: The manuscript "Taking Stock: Can captive species contribute to biodiversity conservation?" addresses a relevant and debated topic in zoo biology and conservation policies. The paper is well-written, clear, and provides a novel regional perspective by assessing both species richness and population abundance within BIAZA institutions. The methodology is generally robust and effectively uses the Shannon Diversity Index to explore ex-situ population dynamics.Major/Minor Concerns (Statistical Clarification)Methodology & Analysis (Anova Assumptions): In lines 222-224, the authors state they used an ANOVA test to analyze native species population sizes across vertebrate classes because homoscedasticity was met (Lavene's test p<0.090). However, since the overarching dataset presented non-normal distribution patterns (as stated for the Mann-Whitney and Kruskal-Wallis segments), please clarify if the normality assumption was verified specifically for the native species subset to validate the use of a parametric test.ConclusionThe manuscript meets most of PLOS ONE’s publication criteria. It reports original research, adheres to ethical standards for metadata usage, and provides full data availability. Addressing the minor statistical detail regarding the ANOVA assumptions will further strengthen the manuscript.

**********

6. PLOS authors have the option to publish the peer review history of their article (what does this mean?). If published, this will include your full peer review and any attached files.

If you choose “no”, your identity will remain anonymous but your review may still be made public.

**Do you want your identity to be public for this peer review?**  For information about this choice, including consent withdrawal, please see our Privacy Policy.

Reviewer #1: No

Reviewer #2: **Yes:**  ACACIA EDUARDA DE JESUS NASCIMENTO

**********

[NOTE: If reviewer comments were submitted as an attachment file, they will be attached to this email and accessible via the submission site. Please log into your account, locate the manuscript record, and check for the action link "View Attachments". If this link does not appear, there are no attachment files.]

To ensure your figures meet our technical requirements, please review our figure guidelines: https://journals.plos.org/plosone/s/figures

You may also use PLOS’s free figure tool, NAAS, to help you prepare publication quality figures: https://journals.plos.org/plosone/s/figures#loc-tools-for-figure-preparation.

NAAS will assess whether your figures meet our technical requirements by comparing each figure against our figure specifications.

# Author comment: Author response to Decision Letter 1

**DOI:** 10.1371/journal.pone.0354602.r002

22 Jun 2026

Dear Reviewers,

Firstly, thank you for taking the time to read my paper and providing me with your comments and suggestions. I have addressed all reviewer recommendations and below, provide detail of the actions taken.

- Reviewer 1, comment 1- “A distinction might be useful here. Housing threatened species only makes conservation sense if they are used for research or breeding and release that benefits wild populations. Holding them alone does not mean any conservation action is actually taking place. And in cases where the ex-situ populations of animals are not sustainably managed, it may actually be more ethical to house non-threatened species, since removal from the wild is less likely to impact species’ status”.

- Response (line 86-90) – Clarification provided over the holding of threatened species and their contribution to conservation. Three new references provided (23-25).

- Reviewer 1, comment 2 “I don’t dispute that covering 92% of BIAZA members should provide a good overview of the status of BIAZA collections. However, did the authors identify any biases in the institutions they weren’t able to access? For instance, if that 8% covered mostly small, regional zoos, then they might be missing out on collections of regionally important species. Alternatively, if it was a random sampling that were missed, then it would be less likely to introduce biases. This extra information would be helpful for readers to assess the quality of the sample”.

- Response (line 163-165) – Provided the number of institutions missing from the analysis along with size of institution. However, to protect Institutional anonymity and consistency with the rest of the document, no names of intuitions are provided. Reference 43 added.

- Reviewer 1, comment 3 - “Table 1: the figure capture is missing a description for “DD”, which presumably is data deficient.”

- Response (line 250) – Added missing Data Deficient (DD) criterion to table 1 legend.

- Reviewer 1, comment 4 - “The cause and effect here is hard to disentangle. Yes, a high proportion of BIAZA’s amphibian species are threatened, but might that not simple be because amphibians are more threatened globally than other species (line 89)? It does not mean these species were selected for collection planning because of their status but could be that wild population trends have been so poor that more of the species held have become threatened over time”.

- Response (378-381) – Provided clarifying sentence around the reason for high number of threatened amphibians with the provision of reference 27.

- Reviewer 2, comment 1 In lines 222-224, the authors state they used an ANOVA test to analyse native species population sizes across vertebrate classes because homoscedasticity was met (Lavene's test p<0.090). However, since the overarching dataset presented non-normal distribution patterns (as stated for the Mann-Whitney and Kruskal-Wallis segments), please clarify if the normality assumption was verified specifically for the native species subset to validate the use of a parametric test.

- Response (line 224-225) – Added line stating the use of parametric testing specifically for native species subset.

- During the resubmission a small error was noticed with figure 3c. Author changed fish representation from 6 species to 3 as 3 of the species were categorised as DD rather than a threatened categorisation. The proportional representation of fish was changed from 10% to 5% and the number of individuals from 39 to 18. On line 326-327 The necessary changes were made to the MIN, MAX, AVERAGE and SD for the number of individuals per species for globally threatened species native to the UK. This minor edit had no change to the statistical analysis or discussion

Thank you once more for the comments regarding this paper and I look forward to finding out the revised outcome.

Kind regards,

Justin Lennon

PhD student

Attachment Submitted filename: Response to reviewers.docx

# Editor report: Decision Letter 1

**DOI:** 10.1371/journal.pone.0354602.r003

**Authors:** Ulrike Gertrud Munderloh

9 Jul 2026

Taking Stock: Can captive species contribute to biodiversity conservation?

PONE-D-26-02051R1

Dear Dr. Lennon,

We’re pleased to inform you that your manuscript has been judged scientifically suitable for publication and will be formally accepted for publication once it meets all outstanding technical requirements.

Within one week, you’ll receive an e-mail detailing the required amendments. When these have been addressed, you’ll receive a formal acceptance letter and your manuscript will be scheduled for publication.

An invoice will be generated when your article is formally accepted. Please note, if your institution has a publishing partnership with PLOS and your article meets the relevant criteria, all or part of your publication costs will be covered. Please make sure your user information is up-to-date by logging into Editorial Manager at Editorial Manager® and clicking the ‘Update My Information' link at the top of the page. For questions related to billing, please contact billing support.

If your institution or institutions have a press office, please notify them about your upcoming paper to help maximize its impact. If they’ll be preparing press materials, please inform our press team as soon as possible -- no later than 48 hours after receiving the formal acceptance. Your manuscript will remain under strict press embargo until 2 pm Eastern Time on the date of publication. For more information, please contact onepress@plos.org.

Kind regards,

Ulrike Gertrud Munderloh, Ph.D.

Academic Editor

PLOS One

Additional Editor Comments (optional):

Reviewers' comments:

# Editor report: Acceptance letter

**DOI:** 10.1371/journal.pone.0354602.r004

**Authors:** Ulrike Gertrud Munderloh

PONE-D-26-02051R1

PLOS One

Dear Dr. Lennon,

I'm pleased to inform you that your manuscript has been deemed suitable for publication in PLOS One. Congratulations! Your manuscript is now being handed over to our production team.

At this stage, our production department will prepare your paper for publication. This includes ensuring the following:

* All references, tables, and figures are properly cited

* All relevant supporting information is included in the manuscript submission,

* There are no issues that prevent the paper from being properly typeset

You will receive further instructions from the production team, including instructions on how to review your proof when it is ready. Please keep in mind that we are working through a large volume of accepted articles, so please give us a few days to review your paper and let you know the next and final steps.

Lastly, if your institution or institutions have a press office, please let them know about your upcoming paper now to help maximize its impact. If they'll be preparing press materials, please inform our press team within the next 48 hours. Your manuscript will remain under strict press embargo until 2 pm Eastern Time on the date of publication. For more information, please contact onepress@plos.org.

You will receive an invoice from PLOS for your publication fee after your manuscript has reached the completed accept phase. If you receive an email requesting payment before acceptance or for any other service, this may be a phishing scheme. Learn how to identify phishing emails and protect your accounts at https://explore.plos.org/phishing.

If we can help with anything else, please email us at customercare@plos.org.

Thank you for submitting your work to PLOS ONE and supporting open access.

Kind regards,

PLOS ONE Editorial Office Staff

on behalf of

Dr. Ulrike Gertrud Munderloh

Academic Editor

PLOS One
